# Meibomian gland features in a Norwegian cohort of patients with primary Sjögren´s syndrome

**DOI:** 10.1371/journal.pone.0184284

**Published:** 2017-09-08

**Authors:** Xiangjun Chen, Øygunn Aass Utheim, Jiaxin Xiao, Muhammed Yasin Adil, Aleksandar Stojanovic, Behzod Tashbayev, Janicke Liaaen Jensen, Tor Paaske Utheim

**Affiliations:** 1 Department of Ophthalmology, Vestre Viken Hospital Trust, Drammen, Norway; 2 Department of Ophthalmology, Oslo University Hospital, Oslo, Norway; 3 Department of Medical Biochemistry, Oslo University Hospital, Oslo, Norway; 4 Faculty of Medicine, University of Oslo, Oslo, Norway; 5 Department of Ophthalmology, University Hospital of North Norway, Tromsø, Norway; 6 Department of Oral Surgery and Oral Medicine, Faculty of Dentistry, University of Oslo, Oslo, Norway; 7 Department of Oral Biology, Faculty of Dentistry, University of Oslo, Oslo, Norway; University of Reading, UNITED KINGDOM

## Abstract

**Purpose:**

To assess the tear film and meibomian gland (MG) features in a Norwegian cohort of patients with primary Sjögren´s syndrome (pSS) and in age- and gender-matched control subjects.

**Methods:**

Thirty-four female patients with pSS (age 52.9±11.9 years) and 32 female control subjects (age 49.0±11.5 years) were recruited. After completion of Ocular Surface Disease Index (OSDI) questionnaire and McMonnies Dry Eye Questionaire, participants underwent measurements of tear osmolarity, tear break-up time (TBUT), ocular surface and corneal staining, Schirmer I test, corneal sensitivity, MG expressibility evaluations, and lid margin morphology examination using slitlamp microscopy. Non-contact infrared meibography images were assessed by computer-assisted analysis. The MG loss, calculated as (tarsal area-MG area)/tarsal area, was evaluated in both upper (UL) and lower lids (LL).

**Results:**

Compared to the control group, pSS patients demonstrated higher MG loss in both UL (33.8±13.2% vs. 24.4±8.5%, *p< 0*.*01*) and LL (52.5±15.7% vs. 43.0±9.6%, *p*<0.05), as well as higher lid abnormality score (0.8±0.8 vs. 0.2±0.6, *p*< 0.01). Furthermore, pSS patients showed higher OSDI and McMonnies questionnaire scores, elevated osmolarity, shorter TBUT, shorter blink interval, less wetting in Schirmer I test, more ocular surface staining and more corneal staining. MG loss in UL correlated negatively with TBUT (r = -0.386, *p* = 0.029) in the pSS group, whereas MG loss in LL correlated negatively with TBUT (r = -0.380, *p* = 0.035) in the control group.

**Conclusions:**

Significantly elevated dry eye symptoms and signs were found in the pSS group compared with the control group, which might be attributed to both decreased aqueous tear production and increased tear evaporation.

## Introduction

Primary Sjögren´s syndrome (pSS) is a systemic, progressive, autoimmune disorder characterized by lymphocytic infiltration of exocrine glands and epithelia in multiple sites. The involvement of lacrimal and salivary glands results in dry eye and dry mouth [1]. Meta-analysis of six studies reporting incidence rate of Sjögren´s syndrome (SS) revealed geographic heterogeneity with a higher incidence rate in Asia (6.6 per 100 000 person/years) compared to that in Europe (IR between 3.9 and 5.3 per 100 000 person/year) and US (IR of 3.9 per 100 000 person/year) [2]. Primary Sjögren´s syndrome affects 9–10 females per male, and the mean age at diagnosis was 50–60s [2, 3].

Dry eye disease (DED), as defined by the 2007 International Dry Eye Workshop (DEWS), is “a multifactorial disease of the tears and ocular surface that results in symptoms of discomfort, visual disturbance, and tear film instability with potential damage to the ocular surface” [4]. It is usually classified into two major categories: aqueous tear-deficient dry eye due to failure of lacrimal tear secretion, and evaporative dry eye due to excessive water loss from the exposed ocular surface [4]. There is often an overlap in the occurrence of these two types of dry eye. Female sex and aging increase the risk for DED [5, 6]. A study by Wan et al. showed that among patients with DED those suffering from pSS have higher prevalence and severity of depression [7].

Dry eye in pSS has been mainly associated with decreased aqueous tear production caused by the lacrimal gland involvement in the autoimmune process [4]. However, a study by Goto et al. showed that tear evaporation rate measured by ventilated chamber system was significantly higher in aqueous tear-deficient dry eye in patients with SS compared with non-SS aqueous tear-deficient dry eyes [8]. Furthermore, the incidence of meibomian gland dysfunction (MGD) has been reported to be higher in patients with SS compared to that of subjects without SS [9–11]. Meibomian glands (MGs) are specialized sebaceous glands, which secrete lipids into tears, forming an oily layer of the pre-ocular tear film to prevent excessive evaporation of tears. Therefore, a defective tear film lipid layer caused by MGD might, in part, contribute to the dry eye problem in patients with SS by the increase in tear evaporation.

Meibography allows direct observation of the morphology of the MGs and it is the only method available for the assessment of partial glands or total MG dropout in the tarsal plate [12]. Some studies have found that MG atrophy correlates with functional dry eye parameters such as tear film break-up time (TBUT), expressible MGs and lipid layer thickness [13–15].

There are currently few studies on MGD in SS. Transillumination meibography has been used to assess the MG atrophy in patients with SS [9, 11]. However, only the lower eyelids were evaluated. Using non-contact infrared meibography, Menzies and colleagues [10] found that MG dropout score was significantly higher for the SS group compared to control subjects. In their study, however, only 11 patients with SS were included, and the total MG dropout score obtained by summing the upper and lower eyelids was used for analysis. MG loss may be different in the upper and lower eyelids [15]; therefore, investigating the eyelids separately may help to discover syndrome specific characteristics of MGD in patients with pSS. Furthermore, MG morphological assessments, such as thickness, length, and density may offer additional information [15, 16]. In the current study, non-contact infrared meibography was evaluated in a Norwegian cohort of patients with pSS in comparison with age- and gender-matched controls, and the results of MG loss were correlated to other clinical dry eye tests.

## Materials and methods

### Study subjects

This paper is part of a larger study where interdisciplinary, comprehensive oral and ocular evaluation of patients with pSS was performed. Thirty-four female patients diagnosed with pSS (average age, 52.9±11.9 years; range, 32–72) and a control group of 32 age-matched healthy female subjects (average age, 49.0±11.5 years; range, 32–79) were recruited in the current study. The pSS patients were classified by a rheumatologist according to the American-European Consensus Group 2002 revised criteria [1]. Briefly, the rules for classification require the presence of at least 4 of the 6 criteria items or 3 of the 4 objective criteria items. The six criteria items include subjective and objective ocular dryness; subjective and objective evidence of salivary gland involvement as determined by unstimulated whole salivary flow, sialography, or salivary scintigraphy; presence of Sjögren-specific antibodies to Ro(SSA), or La(SSB) antigens, or both; and positive minor salivary gland biopsy. In this study, a mandatory criterion for the patient inclusion was positive Sjögren-specific antibody serologic results. None of the participants had been treated by punctal plug insertion or by surgical occlusion.

All participants in the control group had a negative history of dry eye/dry mouth complaints, presence of systemic disorder with ocular involvement, any ocular disease, and previous surgery or use of medication that may affect lacrimal and salivary glands secretion.

Regional Medical Ethical Committee of South-East Norway approved the study (2015/363). Written informed consent was obtained from all the participants, and the study was performed in compliance with the tenets of the Declaration of Helsinki.

### Clinical evaluation

The subjects were required to not use topical eye drops within two hours prior to the clinical examination. Each subject was asked to complete the Ocular Surface Disease Index (OSDI) questionnaire[17] (Allergan Inc., Ivine, CA) and a modified McMonnies Dry Eye Questionnarie[18, 19] for standardized evaluation of the symptomatology of dry eye.

After completion of the questionnaires, study subjects underwent a detailed ophthalmic examination in a predefined sequence as follows: the lower tear meniscus height measurement with Keratograph 5M (Oculus, Wezlar, Germany) [20], tear film osmolarity measurement using TearLab Osmolarity System (TearLab Corp, San Diego, CA), count of blink frequency, tear quality evaluation using TBUT after instillation of 5 μl 2% fluorescein sodium, corneal and bulbar conjunctival staining with fluorescein recorded according to the Oxford scoring scheme (range of ocular surface staining: 0–15; range of corneal staining: 0–5) [21], Schirmer I test without topical anesthesia, assessment of corneal sensitivity by using the Cochet-Bonnet nylon thread esthesiometer, and biomicroscopic examination of the ocular adnexa and anterior segment. Ocular Protection Index (OPI) [22] was calculated as the ratio of the TBUT divided by the blink interval. An OPI value of less than 1 implies that tear film break-up occurs within the blink interval.

**Fig 1:**
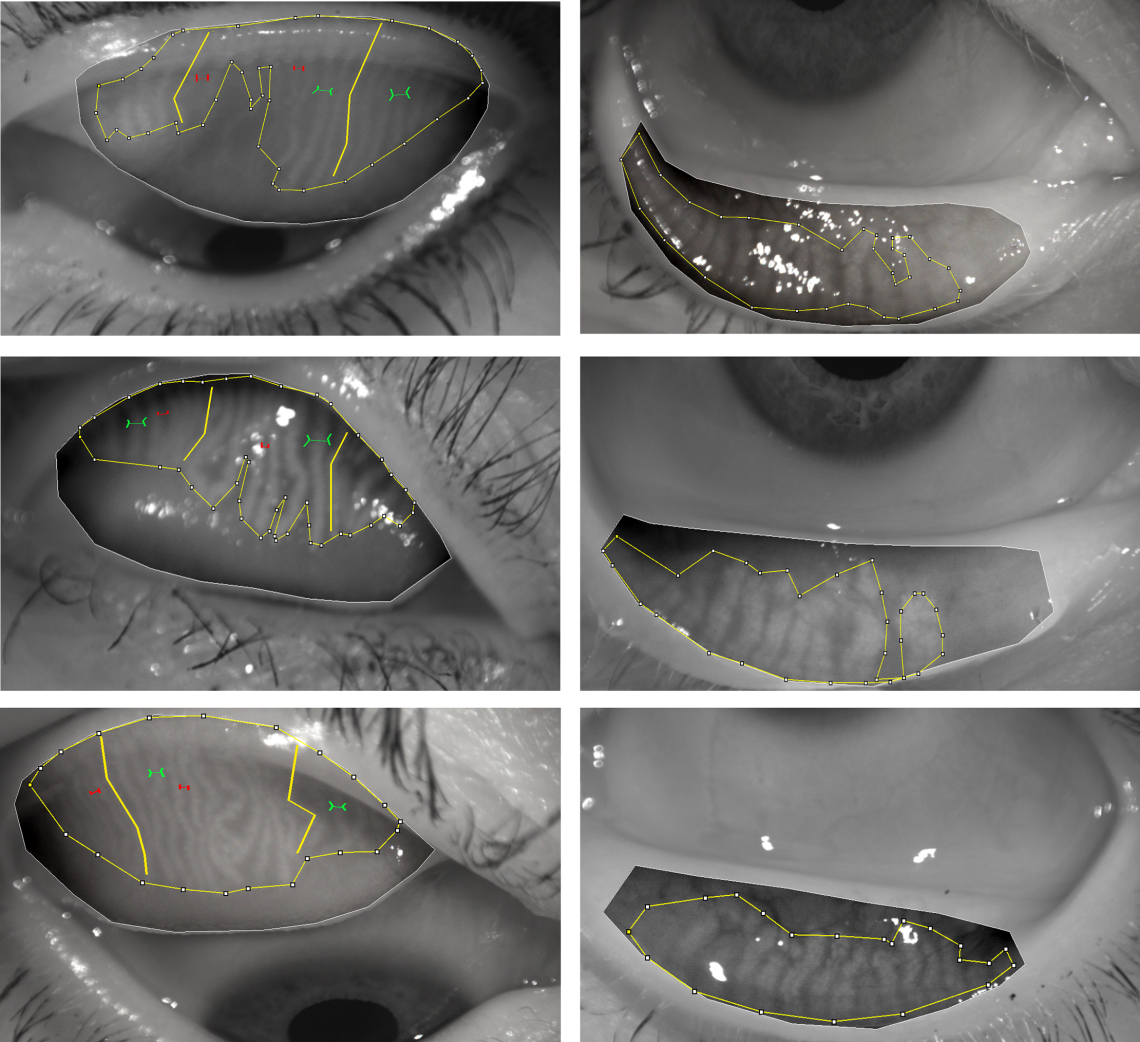
Computer-assisted analysis of meibomian gland (MG) morphology. The MG length, thickness, and gap are marked as thick yellow line, green line, and red lines, respectively. The photos in the left column show MG loss in the upper eyelids of three eyes of 58.2%, 57.8%, and 67.9%, from top to bottom, respectively, whereas the photos in the right column show MG loss in the lower eyelids of the same eyes of 58.6%, 47.1%, and 64.9%, from top to bottom, respectively.

The eyelid margins and the meibomian gland secretions were evaluated on a slit lamp. Lid margin abnormalities were scored as 0 (absent) or 1 (present) for the following parameters at the lower lids: lid margin telangiectasia, posterior lid margin hyperemia, and irregular lid margin. Assessment of the MGs was conducted by application of light pressure using cotton tips on central five MGs of the lower eyelid. Expressibility was recorded as the number of the MGs with meibum secretion under the pressure. The quality of the meibum that was secreted from each gland was graded as following: 0, clear; 1, cloudy; 2, cloudy with particles; and 3, toothpaste like. To avoid giving a false low value in cases where many MG orifices were plugged, the meibum quality value was averaged with the number of glands that expressed meibum per eyelid. At the end, meibography images were obtained using the non-contact infrared camera system Oculus Keratograph 5 after eyelids were everted. The MG loss, calculated as (tarsal area-MG area)/tarsal area, was evaluated by a masked observer using computer-assisted analysis with ImageJ software (National Institutes of Health, NY, USA). It is presented in a 0–100% scale, in which 0% means no MG loss, whereas 100% represents complete MG loss (Fig 1). The computer-assisted MG loss was assessed separately for the upper (UL) and lower eyelids (LL). Additional computerized morphological analyses of the MGs were performed on the UL only. The MG thickness was measured on the three most representative glands across the entire eyelid, and the MG length was calculated by measurement of the three most prominent MGs, whereas the density of the MG area was assessed by measuring the gap between two adjacent MGs at three different locations of the eyelid (Fig 1). A larger gap-measurement value indicates a lower density. The averages of three measurements in MG thickness, length and density were calculated and used in further statistical analyses.

Percentage of pathological findings in each group was calculated. Due to a lack of agreement among the established DED diagnostic criteria, the following cut-off values were adopted in the current study: OSDI questionnaire score ≥ 15 [17], McMonnies questionnaire score ≥ 15 [23], TMH≤ 0.1 mm, osmolarity> 316 mOsm/L [24], TBUT≤ 5 seconds [25], ocular vital staining≥ 3, corneal vital staining≥ 1, Schirmer I test ≤ 5 mm in 5 minutes [26, 27], MG expressibility< 5, OPI< 1, and lid abnormality score > 0.

### Statistical analysis

The values from averaging findings in both eyes of each participant were used for analysis. The statistical analysis was performed with commercial software SPSS for Mac, version 23 (SPSS Sciences, Chicago, IL). All the data are expressed as mean ± standard deviation (SD). The normal distribution of variables was verified by the Shapiro-Wilk test. General linear model was used to adjust factor of age in inter-group comparison. Binomial variables were compared with χ^2^ test. Correlations between variables were undertaken by using Pearson or Spearman rank correlation analysis, depending on the distribution of the variables. A *p* value of < 0.05 was considered to be statistically significant.

## Results

### Dry eye tests

A statistically significant difference was found between pSS and control group in most of the parameters studied (Table 1). Compared to the control group, pSS group demonstrated higher OSDI and McMonnies scores, higher osmolarity, shorter TBUT, lower OPI, higher blink frequency, less wetting of Schirmer I test, lower TMH, more ocular and corneal staining, and higher lid abnormality score. No statistically significant difference was found between pSS and control groups with regard to corneal sensitivity, MG expressibility, or meibum quality per gland.

**Table 1:** Dry eye tests in subjects with and without primary Sjögren´s syndrome.

Parameters	N	Control group	N	pSS group	*P* value
**Age (years)**	32	49.0±11.5	34	52.9±11.9	0.348
**Range**		32–79		32–72	
**OSDI**	32	4.8±7.5	34	34.8±19.2	0.000*
**Range**		0.0–39.6		2.3–86.4	
**McMonnies**	32	4.1±2.0	34	17.6±3.8	0.000*
**Range**		0–8		11–27	
**Osmolarity (mOsm/L)**	31	319.7±15.8	30	334.8±21.5	0.003*
**Range**		295.5–358.0		295.5–374.5	
**TBUT (sec)**	32	5.4±3.3	34	2.4±2.6	0.000*
**Range**		1.0–15.0		1.0–15.0	
**OPI**	30	1.9±1.4	32	1.0±0.8	0.003*
**Range**		0.2–5.6		0.2–4.2	
**Blink frequency per minute**	30	23.1±12.2	32	32.1±13.3	0.009*
**Range**		3–50		7–55	
**Schirmer I (mm/5min)**	29	16.2±11.6	30	4.8±4.0	0.000*
**Range**		0.0–35.0		0.0–14.5	
**TMH (mm)**	29	0.22±0.08	34	0.15±0.06	0.000*
**Range**		0.10–0.41		0.08–0.35	
**Ocular staining**	32	0.8±1.2	34	3.9±2.3	0.000*
**Range**		0.0–5.5		0.0–10.0	
**Corneal staining**	32	0.3±0.5	34	1.8±1.1	0.000*
**Range**		0.0–2.0		0.0–4.0	
**Corneal sensitivity (mm)**	20	58.8±2.9	22	58.1±3.9	0.596
**Range**		50.0–60.0		45.0–60.0	
**MG expressibility**	31	3.4±1.4	32	3.1±1.3	0.630
**Range**		0.5–5.0		0.0–5.0	
**Meibum quality per gland**	31	0.2±0.4	31	0.1±0.4	0.439
**Range**		0–15		0–2	
**Lid abnormality score**	27	0.2±0.6	31	0.8±0.8	0.007*
**Range**		0.0–2.0		0.0–3.0	

OSDI = Ocular Surface Disease Index questionnaire; TBUT = tear film break-up time; TMH: tear meniscus height; OPI = ocular protection index; MG = meibomian gland. Values marked with * represent statistically significant inter-group difference adjusted for age using general linear model.

### Percentage of eyes presenting pathological values

**Fig 2:**
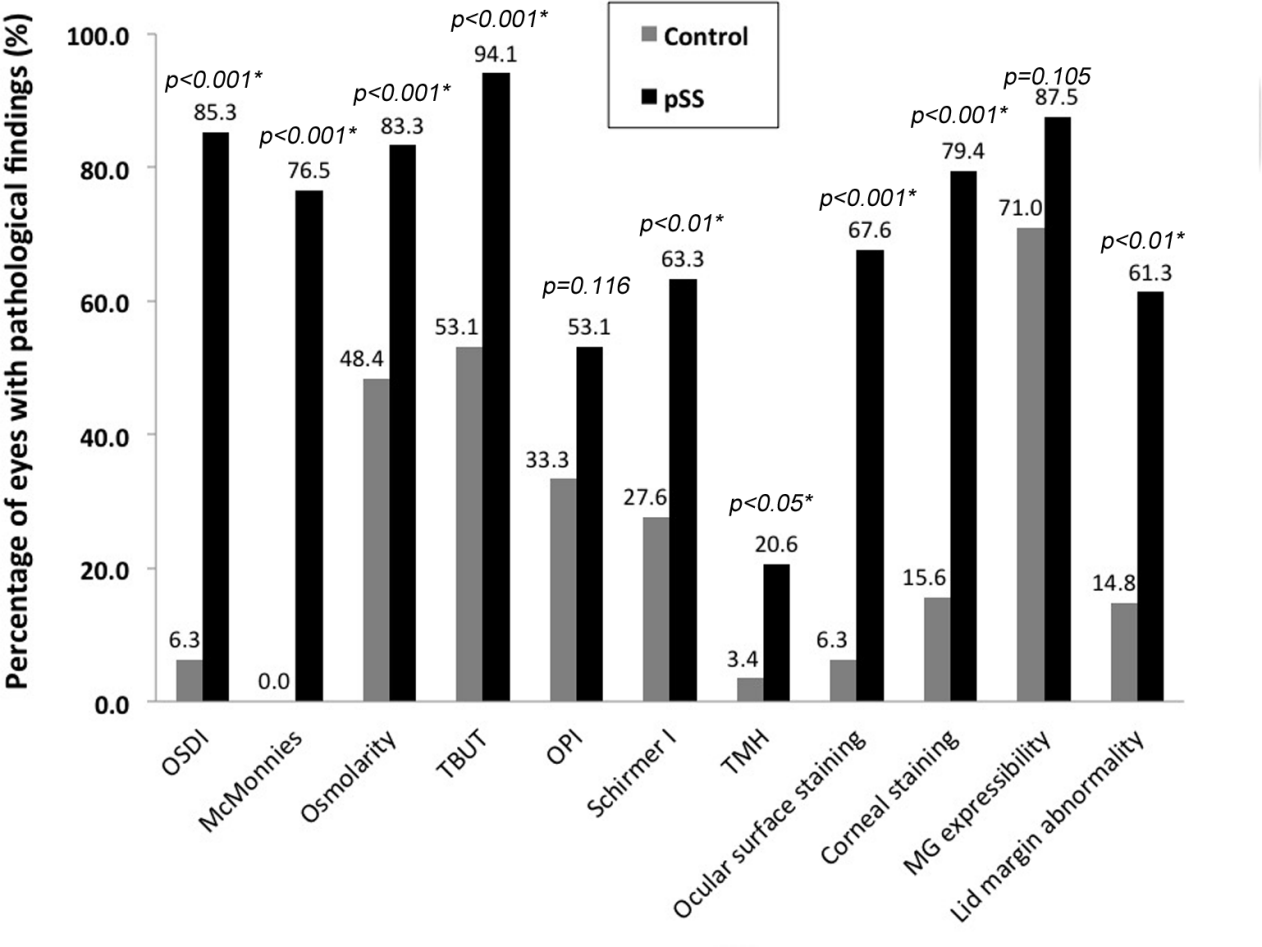
Percentage of abnormal results of the dry eye diagnostic tests obtained in primary Sjögren´s syndrome group and control group. TBUT = tear film break-up time; TMH = tear meniscus height; OPI = ocular protection index; MG = meibomian gland. Values marked with * represents statistically significant inter-group differences using χ^2^ test.

In pSS group, pathologically high OSDI questionnaire score, high McMonnies questionnaire score, high osmolarity, decreased TBUT, low Schirmer I test value, low TMH, pathological ocular surface staining and corneal staining, and abnormal lid margin features were found in 85%, 76%, 83%, 94%, 63%, 21%, 68, 79%, and 61% of the cases, respectively. These percentage values were higher than in control group, in which the respective values were 6%, 0%, 48%, 53%, 28%, 3%, 6%, 16%, and 15% (*p*< 0.05 in call cases) (Fig 2). Low OPI was found in 53% and 33% of the cases in pSS and control groups, respectively (*p* = 0.116). Abnormal MG expressibility was present in 88% of the pSS patients and in 71% of the control group (*p* = 0.105).

### Morphology of meibomian glands and its correlation with other clinical measurements

The MG loss in both eyelids was significantly higher in the pSS group (Table 2). Also, the incidence of MG loss more than 50% was higher in the pSS group than in the control group (9.3% vs. 0% in the UL; 51.5% vs. 22.6% % in the LL). In the pSS group, the MG length in the UL correlated negatively with age (Spearman´s correlation r = -0.402, *p* = 0.022), and MG loss in UL correlated negatively with TBUT (Spearman´s correlation r = -0.386, *p* = 0.029). In the control group, MG loss in LL correlated positively with age (Pearson´s correlation r = 0.454, *p* = 0.010), correlated negatively with TBUT (Spearman´s correlation r = -0.380, *p* = 0.035), and negatively with MG expressibility (Spearman´s correlation r = -0.568, *p* = 0.001). MG loss in UL correlated negatively with MG expressibility (Spearman´s correlation r = -0.421, *p* = 0.020) and McMonnies score (Pearson´s correlation r = -0.382, *p* = 0.034). MG gap correlated negatively with McMonnies score (Pearson´s correlation r = -0.386, *p* = 0.032). MG length correlated positively with OSDI score (Spearman´s correlation r = 0.439, *p* = 0.014).

**Table 2:** The morphology of meibomian glands in subjects with and without primary Sjögren´s syndrome.

Parameters	N	Control group	N	pSS group	*P value*
**MG loss_UL, %**	31	24.4±8.5	32	33.8±13.2	0.004*
**Range**		9.6–45.2		19.6–85.2	
**MG loss_LL, %**	31	43.0±9.6	33	52.5±15.7	0.012*
**Range**		28.5–62.4		27.1–89.3	
**MG thickness, Image J unit**	31	18.4±3.0	32	19.5±3.8	0.224
**Range**		12.3–23.7		9.7–26.6	
**MG length, Image J unit**	31	290.3±47.3	32	277.8±49.2	0.476
**Range**		198.2–378.9		138.2–348.0	
**MG gap, Image J unit**	31	16.3±2.4	32	15.7±1.8	0.235
**Range**		11.2–22.1		10.5–18.1	

MG = meibomian gland; UL = upper lid; LL = lower lid. Values marked with * represent statistically significant inter-group difference adjusted for age using general linear model.

## Discussion

The current study demonstrated a higher score of subjective dry eye symptoms, elevated tear film osmolarity, less stable tear film, decreased tear production, more meibomian gland atrophy, as well as more lid margin abnormalities in a Norwegian cohort of patients with pSS compared to that of the age- and gender-matched control group.

The ocular surface tear film consists of mucin, aqueous, and lipid layers. The mucin layer is secreted by the goblet cells and the ocular surface epithelium, aqueous components are secreted from the lacrimal gland, and the lipid layer is formed by MG secretion (meibum), which acts to prevent excessive tear evaporation. The tear film spreads across the ocular surface by blinking, and the TBUT value is considered an index of tear film instability. When tear film break-up occurs within the blink interval, it is assumed to give rise to local drying and hyperosmolarity of the exposed surface, to surface epithelial damage, and to a disturbance of glycocalyx and goblet cell mucins. The latter consequently exacerbates the tear film instability as part of a vicious circle of events [28].

Decreased aqueous production is known to be a major component of pSS-related ocular surface abnormality [4]. As shown in our study, a lower value of Schirmer I test and TMH in patients with pSS were found, compared to that of age- and gender-matched control group, and 63% of the pSS patients had Schirmer I test≤ 5mm. It should be noted, however, that patients with SS often exhibit more severe changes in the ocular surface than do dry eye patients without SS [9, 29]. Tear evaporation rate was reported to be significantly higher in aqueous tear-deficient dry eyes in patients with SS compared with non-SS aqueous tear-deficient dry eyes [8]. Therefore, it is suggested that the combination of aqueous deficient dry eye and evaporative dry eye have amplified the dry eye state in SS patients.

Embedded in parallel rows in the tarsal plates of the eyelids, there are approximately 30–40 of MGs in the UL and 20–30 MGs in the LL [30]. Meibum is secreted through the orifices located on the lid margin into the marginal reservoirs and then spread over the pre-ocular tear film in the up-phase of each blink. Meibomian gland dysfunction is the major cause of evaporative dry eye. The clinical key signs of MGD include MG dropout (the loss of acinar tissue detected by meibography), altered MG secretion, and change in lid morphology.[31] In the normal population, hyposecretion of meibum and MG dropout are associated with aging [16, 32], which is in accordance with our finding that the MG loss in LL correlated positively with age in the control group.

Meibomian gland is an androgen target organ, and androgen deficiency is a risk factor for the development of MGD.[33, 34] Women with SS have been shown to be androgen-deficient[35]. Accordingly, in the present study, we found that subjects with pSS displayed a higher degree of MGD than the controls, evident from higher MG loss and a higher incidence of lid margin abnormalities. Surprisingly, no statistically significant difference in MG expressibility and meibum quality per gland was detected between the groups in the current study. These findings may support Jester et al.´s hypothesis that the key factor in clinical MGD is the defects in MG acinar differentiation and function leading to gland atrophy, as opposed to a mechanism involving duct hyperkeratinization leading to obstruction, dilation and disuse atrophy[36]. Moreover, the pathogenesis of MGD in patients with and without SS may differ. Using laser-scanning in vivo confocal microscopy to evaluate morphologic changes in MGs, discernible patterns of MG abnormalities in SS and non-SS MGD have been found [11]. In patients with non-SS MGD, the increased diameters of acinar units and orifices and high-reflective secretion could be attributed to qualitative changes of the MG secretion and to subsequent MG obstruction. In contrast, less acinar dilatation, lower secretion reflectivity, and decreased orifice diameter were detected in patients with SS, suggesting a minor role for the obstructive pathogenetic mechanism [11]. In addition, the SS patients demonstrated higher inhomogeneous appearance of the interstice of the acinar unit compared to non-SS MGD patients and healthy subjects, which was interpreted to represent inflammation in the eyelid margin and tarsal plate.

Using non-contact infrared meibography, Menzier and associates [10] reported higher MG dropout score in patients with SS compared to control subjects without dry eye. However, unlike the current study, the MG dropout score in their study was a sum of the scores for the UL and LL. The relative contribution of the glands in the UL and the LL is unknown. Although the MGs in the LL are wider than the UL [37], there are a greater number of MGs in the UL than in the LL. Further, the individual MGs are longer in the UL than in the LL [30, 38, 39]. The contribution of meibum by the UL might thus be greater than the LL [30]. Therefore, we separately analyzed MG loss for UL and LL. In line with the former studies [15, 16], our results showed higher MG loss in the LL, compared to that of UL. Also, a higher percentage of MG loss in both lids in pSS patients compared to the control group in the current study supports the data from previous studies [9, 11] demonstrating more MG atrophy in the LL in patients with SS compared to the control group. Furthermore, the negative correlation between MG loss in UL and TBUT in the pSS group in the current study is in compliance with findings by Mathers et al. that patients with MG dropout, and especially those with low tear production by Schirmer test, have an increased risk of dry eye developing through increased evaporation [40]. The MG length, thickness and gap in the UL did not show statistically significant difference in pSS patients and control group. It might be caused by the fact that only the three most representative MGs in the UL were chosen to calculate the values. Further studies using more sophisticated evaluation parameters are warranted to elucidate the mechanism of MGD in patients with SS.

Dry eyes, either due to insufficient tear production or excessive tear evaporation, have increased concentration of tear film constituents, as manifested by elevated osmolarity and rapid TBUT [41]. Hyperosmolarity has been shown to provide a pro-inflammatory stress to the ocular surface [42, 43]. Using factor analysis, conjunctival staining with rose bengal and superior corneal staining with fluorescein was found, among 90 clinical characteristics, to account for the greatest variance (14.7%) in patients with pSS [44]. Hyperosmolarity and increased friction associated with the lid movement in the pSS group might have caused defects of the cornea and conjunctiva, leading to higher grade of staining [45, 46]. The epithelial injury caused by dry eye stimulates corneal nerve endings, leading to symptoms of discomfort and increased blinking [4], which may explain higher blink frequency in our pSS group.

The cause of pSS-related ocular surface changes is multifactorial. Besides aqueous deficiency and the MG atrophy, decreased goblet cell density [47] and reduction in expression of MUC 19 and MUC5AC were found in patients with SS. This is consistent with the significant decrease in mucous secretion in these patients [48, 49]. Mucins deficiency may therefore in part explain tear film instability and disruption of the ocular surface homeostasis in SS. Such investigations, however, were beyond the scope of current study.

Although dry eye is usually symptomatic, some studies showed a lack of association between clinical signs and symptoms of DED [50–52]. For instance, a study by Sullivan et al.[52] demonstrated that more than 40% of subjects with clear objective evidence of dry eye disease are asymptomatic. Also, according to TFOS DEWS II epidemiology report[53], prevalence of DED for studies involving symptoms with or without signs ranged from approximately 5% to 50%, while studies where the diagnosis was based primarily on signs reported higher and more variable rates of DED, up to 75% in certain population [54]. Several participants in the control group, although asymptomatic, demonstrated pathological signs in the clinical dry eye tests, thus they could not be regarded as truly “healthy” subjects. However, they may be more representative of the age-matched normal population.

DED impairs the quality of life of patients with pSS; hence, management of DED is important for treatment of pSS. The current study revealed alterations in tear film stability, aqueous tear production, and meibomian gland morphology. Our results indicate that MGD is involved, at least in part, in the pathogenesis of DED in pSS. The knowledge gained in the present study further our understanding of the underlying mechanisms of DED in pSS and may therefore offer clues for improved therapeutic treatment.

## Supporting information

S1 File MGD in pSS and control comparison. (XLSX)
